# NeoSeq: a new method of genomic sequencing for newborn screening

**DOI:** 10.1186/s13023-021-02116-5

**Published:** 2021-11-18

**Authors:** Huaiyan Wang, Yuqi Yang, Lingna Zhou, Yu Wang, Wei Long, Bin Yu

**Affiliations:** 1 Department of Medical Genetics, Changzhou Maternal and Child Health Care Hospital affiliated to Nanjing Medical University, No. 16 Ding Xiang Road, Changzhou, Jiangsu Province China; 2 Department of Neonatology, Changzhou Maternal and Child Health Care Hospital affiliated to Nanjing Medical University, No. 16 Ding Xiang Road, Changzhou, Jiangsu Province China

**Keywords:** Newborn screening, Tandem mass spectrometry, Newborn genomic sequencing, Next-generation sequencing, Multiplex PCR amplicon sequencing assay

## Abstract

**Objective:**

To explore the clinical application of NeoSeq in newborn screening.

**Methods:**

Based on the results obtained from traditional newborn screening (NBS) with tandem mass spectrometry (TMS), three cohorts were recruited into the present study: 36 true positive cases (TPC), 60 false-positive cases (FPC), and 100 negative cases. The dried blood spots of the infants were analyzed with NeoSeq, which is based on multiplex PCR amplicon sequencing.

**Results:**

Overall, the sensitivity of NeoSeq was 55.6% (20/36) in the detection of TPC. NeoSeq detected disease-related genes in 20 of 36 TPC infants, while it could not identify these genes in eight children. Five cases (3.1%) with disease risk were additionally found in the FPC and NC cohorts. There was a significant difference in the diagnostic time between the two methods—10 days for NeoSeq vs. 43 days for traditional NBS.

**Conclusions:**

NeoSeq is an economic genomic screening test for newborn screening. It can detect most inborn errors of metabolism, reduce the rate of false positive results, shorten the porting cycles, and reduce the screening cost. However, it is still necessary to further optimize the panel design and add more clinically relevant genomic variants to increase its sensitivity.

**Supplementary Information:**

The online version contains supplementary material available at 10.1186/s13023-021-02116-5.

## Introduction

It is well known that newborn screening (NBS) is an important public health project. It facilitates the identification of newborns with genetic and/or metabolic diseases as quickly as possible after birth and helps improve the quality of life through early intervention [1]. Since the 1960s, NBS has been officially applied in clinical practice and experienced continuous technological innovation and development. Since 1989, tandem mass spectrometry (TMS) has been used for NBS [2]. With its technical advantages, it can detect more inborn errors of metabolism (IEM) through a single examination [3]. However, as clinical manifestations are complex and highly variable, the diagnosis of IEM often requires other auxiliary examinations, including variant detection of disease-causing genes. Therefore, the application of next-generation sequencing (NGS) following TMS (TMS-NGS) has become the mainstream of newborn disease screening programs [4]. However, there are still various challenges, such as the number of diseases that should be screened. The American College of Medical Genetics (ACMG) identified 29 conditions for screening [5]. Most institutions in China conduct screening for 27 diseases [6, 7]. Diagnosis and intervention are often delayed because of the long turnaround time of NGS testing, and there are still relatively high numbers of false positives. In addition, the high cost of NGS testing cannot be ignored.

Recently, with the rapid advancement of sequencing and the decrease in costs, NGS has helped make great progress in various medical fields. It not only contributes to the diagnosis and targeted treatment of diseases but can also be used in population screening. Additionally, genomic sequencing, including whole genome sequencing (WGS), exome sequencing (ES) or gene panel sequencing, has shown potential utility in newborn screening [8–10]. It may become another technological innovation, after TMS. Currently, the most authoritative and representative projects are the BabySeq Project [11, 12] and North Carolina New-born Exome Sequencing for Universal Screening (NC NEXUS) study [13]. The BabySeq project is a randomization study of newborn genomic sequencing (nGS) using whole exome sequencing (WES). It identified 954 genes from a list of 1,514 gene–disease associations [14] and recently reported the findings from 159 newborns. The results revealed variants that conferred disease in 15/159 (9.4%) of newborns, which could not be identified from the known clinical phenotypes or family histories. Notably, 10 of the newborns were from a healthy population. The study also found various carrier status and pharmacogenomic variants. Similarly, NC NEXUS explored the value of WES in newborn screening based on healthy newborns and clinically diagnosed cases. The study confirmed the positive screening results in 15 of 17 participants with metabolic disorders, and five of 28 individuals in a hearing loss cohort. Additionally, it discovered four cases with positive results, which were not detected using standard NBS [15]. Although both the studies fully demonstrated the advantages and prospects of nGS in new-born screening, there are still many limitations. nGS cannot replace the current NBS that is based on biochemical measurements. Due to the problems associated with experimental technology and genetic counseling, nGS will face a huge challenge. As it needs more timely family follow-up and participation, the ethical issues and privacy issues involved will be more prominent. Furthermore, the high cost can be prohibitive.

Here, we started a new project named “NeoSeq”, which is based on the multiplex PCR amplicon sequencing assay (MTA-Seq). In this study, we explored the clinical application of NeoSeq in newborn screening and compared and analyzed the effects in different newborn populations. We hope to provide an effective method for new-born genomic screening.

## Materials and methods

### Study design and participants

This was a parallel controlled study conducted at the Department of Medical Genetics, Changzhou Maternal and Child Health Care Hospital affiliated to Nanjing Medical University. From January 2014 to June 2021, a total of 157,500 infants underwent NBS via TMS-NGS. Based on the results obtained, three cohorts were recruited into the present study: (1) TMS-NGS true positive cases (TPC), (2) TMS-NGS false positive cases (FPC), and (3) TMS-NGS negative cases (NC). Written informed contents were obtained from the newborns’ parents before screening.

### Sample collection

As described in our previous study [4], dried blood spots (DBSs) were collected from all infants on a 903 filter paper (Wallace Oy, Turku, Finland) after 72 h of birth. Peripheral blood was collected from the parents of positive newborns for experimental validation.

### TMS-NGS-based newborn sequencing

NBS with TMS and target gene detection using NGS were described previously [4]. All DBSs were analyzed with MS/MS using the NeoBase™ Non-derivatized MS/MS Kit (PerkinElmer, Turku, Finland). Infants with positive results were brought in for further assessment, including any clinical manifestations, individualized assistant examination, and gene detection. Targeted sequencing used the extended edition panel of inherited metabolic diseases (Hangzhou, China), including 306 IEM disease-related genes.

### NeoSeq panel design

The NeoSeq panel (Hangzhou Biosan Clinical Laboratory) contained 2,500 variations of 135 pathogenic genes, which correspond to 75 types of newborn common genetic diseases (Additional file 1: Table S1). The panel included diseases of the skeletal system, hematological system, mitochondria, lysosomal storage, immune system, peroxisomal biogenesis, and others. The criteria for diseases and genes to be included in the NeoSeq panel was drawn from the literature [14] and characteristics of common pathogenic genes in the Chinese population [4].

### Genetic screening using the NeoSeq panel

Genomic DNA was extracted from dried blood spots (2 × 8 mm) collected in this study, using an Nucleic Acid Automatic Extraction System (Bioer, China). Multiplex PCR was used to generate DNA libraries using the SLIMamp (StemLoop Inhibition Mediated amplification) method [16]. The quality of libraries was assessed with Agilent Bioanalyzer 2100 (Agilent Technologies, Santa Clara, CA, USA). High-throughput sequencing was performed using Illumina NextSeq 500 (Illumina, San Diego, CA, USA) according to the manufacturer’s instruction.

### Bioinformatic analysis

Raw image files were processed using Bcl to Fastq (Illumina) for base calling and generating raw data. Low-quality sequencing reads were filtered out and the reads were aligned to the NCBI human reference genome (hg19/GRCh37). The minor allele frequencies (MAFs) of all known variants were also reported according to their presence in the dbSNP (http://www.ncbi.nlm.nih.gov/snp), the 1000 Genome Project (http://browser.1000genomes.org), and Exome Aggregation Consortium (ExAC) (http://exac.broadinstitute.org/). Databases such as OMIM (http://www.omim.org), ClinVar (http://www.ncbi.nlm.nih.gov/clinvar), and Human Gene Variant Database (http://www.hgmd.org) were used to determine variant pathogenicity where appropriate. All target variants were subjected to biological effect analysis, which included the use of programs such as SIFT (http://sift.jcvi.org), VariantTaster (http://www.varianttaster.org), PolyPhen-2 (http://genetics.bwh.harvard.edu/pph2), PROVEAN (http://provean.jcvi.org/index.php) to predict whether an amino acid substitution or indel had an important biological effect.

### Sanger sequencing

Sanger sequencing was used to validate variants positively identified by NeoSeq. Genomic DNA extracted from peripheral whole blood or DBS was amplified using specific primers. PCR amplification of variants was conducted using the Phanta Max Master Mix (Vazyme, China). After the purification of PCR products, sequencing analysis was performed with capillary electrophoresis using an ABI Prism 3500XL Genetic Analyzer.

## Results

**Fig. 1:**
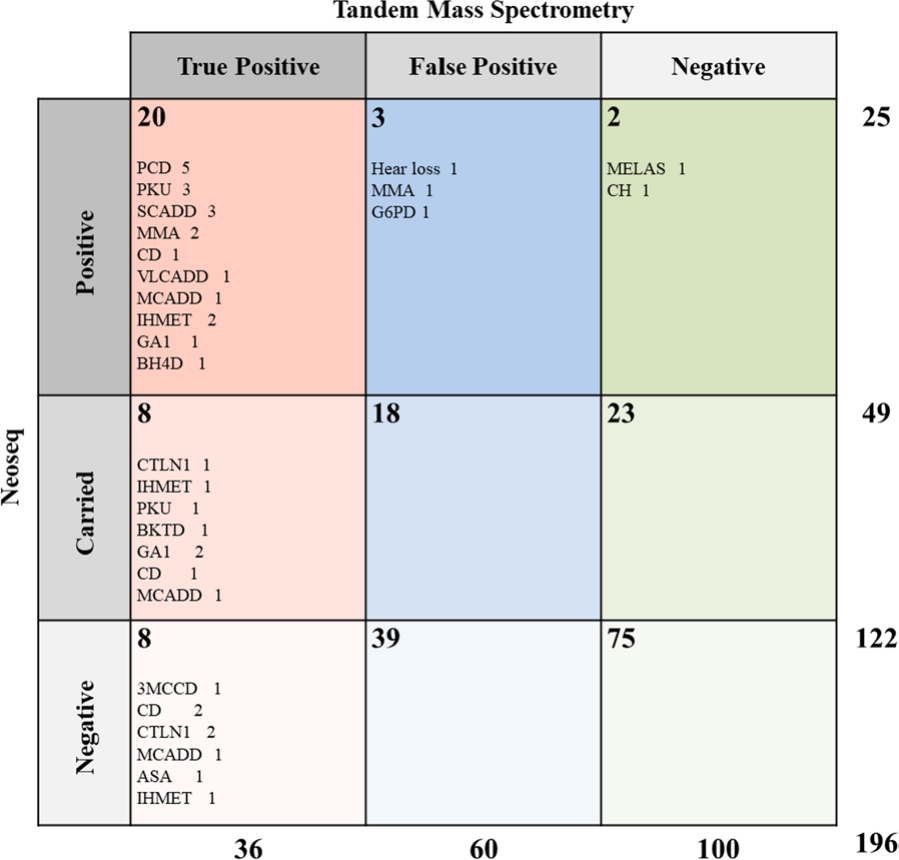
Comparison of tandem mass spectrometry and NeoSeq for newborn screening

**Fig. 2:**
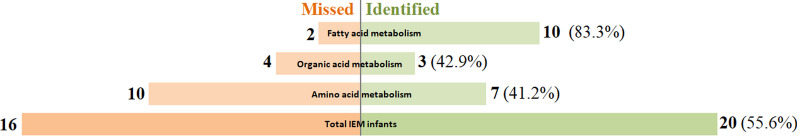
Detection of different inherited metabolic diseases with NeoSeq

Both NeoSeq and TMS-NGS screening were performed on 196 infants in three cohorts, which included IEM confirmed cases and TMS-false positive and TMS-negative babies. Overall, the agreement between the results of the two NBS projects was 55.6% (20/36). NeoSeq screening identified 20 of 36 TPC infants with disease-related genes. In addition, five infants (3.1%) with disease-risk variants were found in the FPC and NC cohorts (Fig. 1). This study mainly focused on three types of genetic metabolic diseases: those related to amino acid metabolism (AAM), organic acid metabolism (OAM) and fatty acid metabolism (FAM). As shown in Table 1 and Fig. 2, the detection rates of NeoSeq were 41.2%, 42.9%, and 83.3% for AAM, OAM, and FAM, respectively.

**Table 1:** Summary of the comparison between TMS and Neoseq

Group	n	TMS-NGS (panel)				Neoseq			Consistency (%)
		NBS result	Recall result	Gene positive	Gene negative	Positive	Inconclusive	Negative	
True positive cases of TMS	36	Abnormal	Abnormal	36	0	20	8	8	55.6
Amino acid metabolism	17	Abnormal	Abnormal	17	0	7	4	6	41.2
Organic acid metabolism	7	Abnormal	Abnormal	7	0	3	3	1	42.9
Fatty acid metabolism	12	Abnormal	Abnormal	12	0	10	1	1	83.3
False positive cases of TMS	60	Abnormal	Normal	–	–	3	0	0	–
Negative cases of TMS	100	Normal	Normal	–	–	2	0	0	–

TMS: Tandem Mass Spectrometry, NBS: Newborn Screening, NGS: next-generation sequencing

Both of the methods detected the genes for the IEM disease (Additional file 1: Table S2). Unexpectedly, eight children with positive IEM results were not identified by NeoSeq, including two cases of citrin deficiency (CD), two cases of citrullinemia type I (CTLN1), and one case each of 3-methylcrotonyl-coenzyme A carboxylase deficiency (3MCCD), medium-chain acyl-CoA dehydrogenase deficiency (MCADD), argininosuccinic aciduria (ASA), and isolated hypermethioninemia (IHMET). Of those, five cases had one pathogenic (P) gene variant and one gene variant of uncertain significance (US), as identified with NGS panel detection following the positive TMS screening. However, the US mutations were not identified using the NeoSeq panel. All the cases were ultimately diagnosed based on clinical manifestations and other diagnostic criteria. Additionally, NeoSeq analysis identified eight out of 36 participants as being carriers of a disease. In most cases, only one pathogenic gene variant was detected by NeoSeq. Other variant is not included included in the NeoSeq panel design due to technical limitations or a very low frequency in population database or literature. For example, one infant (TP022), confirmed as having glutaric acidemia I, was found to have two pathogenic mutations (c.109_110delCA, c.416C>G) in *GCDH*; however, only one mutation (c.416C>G) was detected with NeoSeq. Meanwhile, there was a significant difference in the length of time from testing to diagnosis, between the two methods. With TMS-NGS screening, the participants were able to get results in 20–153 days (median 43 days), although medical interventions were provided in time according to other examinations. In contrast, NeoSeq usually provided results within 7–10 days of testing.

Sixty infants with TMS false-positive results were recruited into this study. Their initial screening values from TMS were abnormal, but all indices returned to normal levels after recall and retesting. NeoSeq was deemed to represent abnormal positive results in three cases (Table 2). One infant diagnosed as having methylmalonic aciduria (MMA) using NeoSeq, was compound heterozygous for a pathogenic variant in the *MMACHC* gene (c.80A>G and c.567dup). His C3 and the ratio of C3/C2 were 7.86 and 0.34 in TMS first screening, and returned to normal levels (C3 = 3.31, C3/C2 = 0.26) after recall. The boy was born full-term (40 weeks) and his birth weight was 3500 g. Unfortunately, he died due to acute hemolysis after 29 days of birth. The disorders of the other two cases were not included in traditional TMS-NGS screening. One participant was hemizygote for a variant (c.1388G>A) in the *G6PD* gene associated with the classic glucose-6-phosphate dehydrogenase deficiency (G6PD). His C3 value was slightly higher at the initial screening. However, the parents refused to bring the baby back and he was lost to follow-up. In another baby, also born full-term, no abnormalities were observed in newborn hearing screening using distortion product otoacoustic emission; however, a heterozygous status for the pathogenic mutation c.547G>A was detected in the *GJB3* gene. This child had been followed up for three months after birth, and no obvious hearing loss was found. In addition, 18 infants were reported as carriers in the cohorts.

**Table 2:** Additional cases found by Neoseq

Group	TMS		Neoseq							Follow-up
	NBS result	Recall result	Disease	Gene	Exon	Nucleotide change	Variant	Mode	Type	
*False positive cases of TMS*										
FP001	C5 = 0.57	C5 = 0.85	Hear loss	*GJB3*	2	c.547G>A	Het	AD	P	
FP002	C3 = 4.15	–	G6PD	*G6PD*	12	c.1388G>A	Hemi	AR	P	
FP003	C3 = 7.86 C3/C2 = 0.34	C3 = 3.31 C3/C2 = 0.26	MMA	*MMACHC*	1,4	c.80A>G c.567dup	Het	AR	P P	
*Negative cases of TMS*										
NC001	normal	–	MELAS	*MTTL1*	/	m.3244A>G	Heteroplasmic	AR	P	
NC002	normal	–	CH	*DUOX2*	13,14	c.1588A>T c.1462G>A	Het	AR	P P	

TMS-NGS (panel): NGS followed Tandem Mass Spectrometry, P: Pathogenic, LP: Likely pathogenic, US: Uncertain significance, AR: Autosomal recessive inheritance, AD: Autosomal dominant inheritance, Het: heterozygotes, Hom: homozygosis. Hemi: hemizygote

Similarly, there were additional findings in the TMS-NGS negative cohorts screened using NeoSeq, although they were beyond the scope of the traditional TMS-based NBS (Table 2). Surprisingly, one girl had a compound heterozygous variant (c.1588A>T and c.1462G>A) in the *DUOX2* gene, which is a well-known pathogenic gene associated with congenital hypothyroidism (CH). Although CH is not included in TMS screening, we also carried out CH screening to find out whether this case was missed. We reviewed the level of neonatal thyroid stimulating hormone (NTSH) in the initial screening and retested its value in the DBSs. The two values obtained were 6.86 and 6.12 mIU/L respectively, which were lower than our cut-off value (9.0). Two months after birth, the girl was hospitalized because of neonatal sepsis, and her serum thyroid functions were examined. The level of TSH was 14.101 mIU/L (reference range: 0.64–6.27); other indicators (T3, T4, FT3, and FT4) were normal. We included her in the follow-up management as a case of hyperthyrotropinemia. Another infant was detected as a heteroplasmic of a pathogenic variant (m.3244A>G) in *MTTL1*, which suggested the risk of mitochondrial encephalomyopathy with lactic acidemia and stroke-like episodes (MELAS). Until now, the girl has not displayed any abnormalities after following up for three months after birth.

**Fig. 3:**
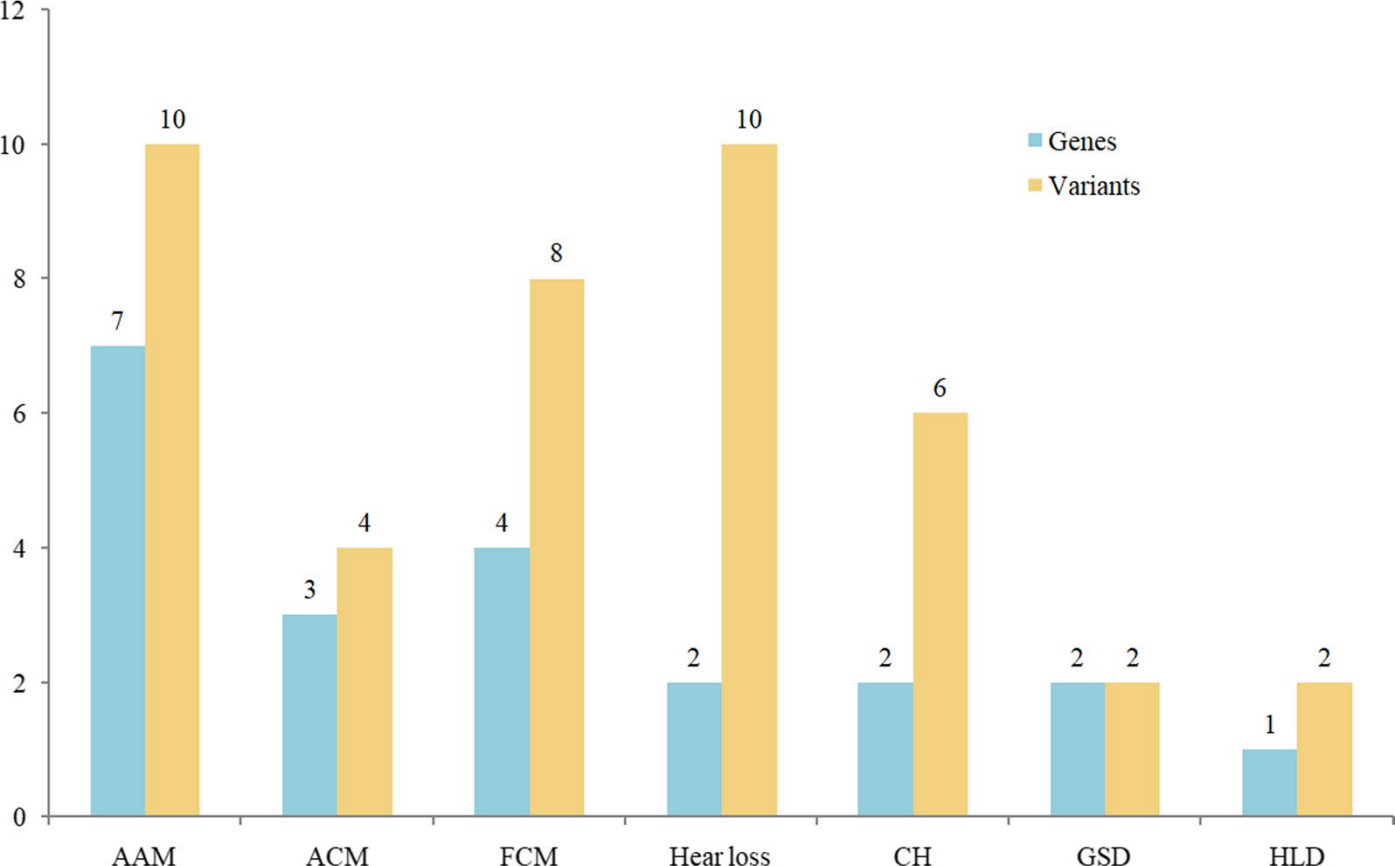
Detection of carrier status in the present study. AAM: amino acid metabolism, OAM: organic acid metabolism. FAM: fatty acid metabolism. CH: congenital hypothyroidism. GSD: glycogen-storager disease. HLD: hepatolenticular degeneration

Additionally, in the present study, we detected a carrier status rate of 25.6% (41/160) in FPC and NC cohorts. Forty-two pathogenic or likely pathogenic variants in 21 genes were found, affiliated with seven kinds of diseases (Fig. 3). The five most commonly identified variants were in the genes *SLC26A4* (5), *GJB2* (5), *DUOX2* (5), *PAH* (3) and *ACADS* (3).

## Discussion

Here, we report a new method of genomic sequencing for newborn screening, called “NeoSeq”, which is based on multiplex PCR amplicon sequencing. As seen from preliminary clinical applications, it has the following advantages: (1) Similar to few nGS studies, NeoSeq can not only detect most of the diseases included in traditional TMS-NGS screening but can also reduce the false-positive rate considerably. (2) It can screen greater number of diseases and provide more genetic health-related information. (3) Current studies usually use the WGS and WES technologies, which are technically difficult to perform and have more complicated experimental procedures than NeoSeq. In addition, WGS and WES have longer reporting cycles and cost significantly more than NeoSeq.

It is well known that TMS has helped to expand the NBS program. NGS technology can be used as the second key step for diagnosis after TMS screening. The combination of both is an enhanced plan for NBS. Currently, more and more countries have adopted TMS-NGS as the primary method for NBS, with good results [4, 17, 18]. With the rapid advancement of sequencing and the subsequent decrease in costs, new-born genomic sequencing (nGS) may become a new milestone in the field of NBS, after TMS, and shows the prospect of broad applications. For example, Bodian’s group presented a comparison of the results of WGS and blood-based NBS from a cohort of 1,696 new-borns. There was generally good agreement between both techniques, with 88.6% true positives and 98.9% true negatives being identified. In addition, the false-positive rate of WGS was far lower than that of the traditional NBS [8]. BabySeq and NC NEXUS projects also reported 60.0–88.0% sensitivity and 100% specificity [12, 15]. Our results showed similar specificity (99.4%), but the sensitivity was relatively lower (55.6%). This may be because we used MTA-Seq as the screening technology rather than WES. Meanwhile, when we designed the NeoSeq panel, we selected criteria of variants in 135 genes as: (1) high frequency in Chinese, Eastern Asian or Asian population; (2) common pathogenic variants in databases, including ClinVar and ClinGen; (3) LOF (Loss of function) variants in Asian population (≥ 10 allele count in Gnomad); and (5) high frequency in local databases. This indicates that NeoSeq precisely reported the definite pathogenic variation; its clinical application is more reliable and genetic consultation is more certain. However, it must be clear that NeoSeq is designed to screen asymptomatic neonates, instead of being a diagnostic test.

Newborn genomic screening is a new research area. To explore the effects of its clinical applications, we conducted a meta-analysis and comparison from a search of the literature (Table 3). Four groups have conducted studies since 2016, which include Newbie Seq [19], NC NEXUS [15], BabySeq [12] and Dale L’ group [8]. All the studies were designed by combining a methodological comparison with the findings from a retrospective cohort. With the exception of Dale L’ group (WGS) [8], the studies used the WES or ES technology. The sensitivity and specificity were 60.0–88.6% and 93.7–100%, respectively. Most studies have reported additional findings beyond the traditional newborn screening. Instead of using WES, we adopted multiplex PCR amplicon sequencing (MTA-Seq) for a number of reasons such as ease of use, simple and standardized procedure, and cost effectiveness. Compared with that of the traditional TMS-NGS screening, the sensitivity of NeoSeq was 55.6% and specificity was 99.4%. Due to different design concepts, there are some differences of the number and variant of genes in TMS-NGS and NeoSeq panel. TMS-NGS is consided as a diagnostic technology, while NeoSeq is designed to population screening. In this study, there may generate a bias when evaluating the detection efficiency of NeoSeq by using the traditional tms-ngs as the standard. Furthermore, the results revealed some interesting additional findings. Importantly, this method can significantly reduce the false-positive rate and the duration of reporting cycle (7–10 days).

**Table 3:** Comparison with other similar studies

Study	Year	Samples	Project	Method	Panel	Sensitivity	Specificity	Additional discovery	Carrier rate
Dale L [1]	2016	1696 infants	–	WGS	163 genes	88.6%	98.9%	G6PD etc	–
Aashish N [2]	2020	1190 805 with IEM 385 with TMS false positives	Newbie Seq	WES	78 genes associated with the 48 IEMs	88.0%	93.7%	–	34.0%
Tamara S [3]	2020	106 17 with IEM 28 with hearing loss cases 61 healthy newborns	NC NEXUS	ES	466 genes	88.0% for IEM 18.0% for hearing loss	100%	OTC deficiency Amilial hypercholesterolemia Actor XI deficiency Arrhythmogenic right ventricular dysplasia	–
Monica H [4]	2021	316 12 with NBS Positive 147 with NBS Negative 127 healthy newborns	BabySeq	ES	954 genes	60.0%	100%	Cardiomyopathy Hereditary breast and ovarian cancer Supravalvular aortic stenosis KBG syndrome Atypical hemolytic-uremic syndrome Glomuvenous malformation Cystinuria Non-syndromic hearing loss Lynch syndrome	-
Our	2021	196 36 with IEM 60 with TMS false positives 100 with TMS negative	Neoseq	MTA-Seq	135 genes related to 75 diseases	55.6% (20/36)	99.4% (159/160)	Hear loss G6PD MELAS CH	26.3% (42/160)

It is yet unclear whether nGS can completely replace TMS-NGS. Current studies agreed that nGS could be used as an important supplement to common blood-based NBS while not completely replacing it. Based on a population screening of 4.5 million infants, the NBSeq project [19] suggested that exome sequencing was not recommended as a first-line method for NBS of IEM. However, it could be used as a secondary test after TMS screens. We support this suggestion. All the studies have shown that the sensitivity of nGS was approximately 55.6–88.6%, which means that some infant diseases diagnosed with traditional NBS may not be detected using nGS. Both complement each other in order to achieve the best effect. Notably, in the present study, five out of eight ill infants missed by NeoSeq only had one pathogenic variant, while the other variant was of uncertain significance. These variants are not reported in NeoSeq for these reasons: (1) The variant is not included in the design due to technical limitations or a very low frequency in population database or literature. (2) The variant is of unknown significance. We are following these children closely. In fact, up to now, these children have no serious clinical manifestations and no special treatment. We just managed them as positive children and strengthened the health care in childhood. On the other hand, is it necessary to screen for these diseases in NBS? It is a scientific problem worthy of discussion [20]. Many countries are constantly optimizing the disease spectrum detected by NBS [4].

It is certain that the advantages of nGS primarily include fewer false-positive results, accurate diagnosis and distinction of disorders, and more useful information for newborn life. Due to the high sensitivity of TMS, the high false-positive rate and low positive predictive value of TMS are always a problem, particularly for some special populations such as premature infants [21]. Additionally, the technique is easily affected by external interference [22]. The high false-positive rate will bring a large number of healthy infants to be excessively recalled, which will not only burden the medical service but also result in psychological burden to the parents [23]. Some researchers even began to question whether TMS should be used to expand NBS [24]. In the present study, three of 60 infants with TMS positive results might have the risk of disease. Therefore, as a supplementary method of TMS screening, nGS can effectively reduce the false-positive rate.

Currently, three methods are primarily used in new-born genomic sequencing: WGS, ES, or gene panel sequencing. However, there are still some doubts whether new-born WGS/WES should be used routinely in clinical applications [25]. They are difficult to popularize due to the complexity of project technology and cost involved. Therefore, they may be not suitable for screening projects. At the same time, they can provide a plethora of genetic information, which could bring great challenges to clinical genetic counselling and also involve a lot of ethical problems. NeoSeq, reported here, is based on multiplex PCR amplicon sequencing (MTA-Seq). This technology is considered a simple, customizable, and targeted sequencing method, which is conducive to the wide application of high-throughput sequencing, such as genome diagnosis, population genetic analysis and so on [26]. Recently, the Yang group [27] applied it to screen genetic hearing loss variants in newborns, demonstrating 100% sensitivity and specificity. We tried to use it to screen 75 kinds of inborn disorders. The results were quite satisfactory. At the same time, most of the experimental detection could be completed in one week, and the cost was only one fifth of that of WES.

In conclusion, NeoSeq is an economic genomic screening test for newborn screening. It can detect most inborn errors of metabolism, reduce the rate of false positive results, shorten the porting cycles, and reduce the screening cost. However, it is still necessary to further optimize the panel design and add more clinically relevant genomic variants to increase its sensitivity.

## Supplementary Information

**Additional file 1. Table S1.** The list of diseases and genes in Neoseq project. **Table S2.** Results of Neoseq in the infants with IEM after TMS screening.

## Data Availability

The questionnaire and datasets used are available from the corresponding author on request.
