# Supplementary material for: NeoSeq: a new method of genomic sequencing for newborn screening
Source: Orphanet J Rare Dis. 2021 Nov 18;16:481. doi: 10.1186/s13023-021-02116-5 (PMC8600711; doi:10.1186/s13023-021-02116-5)
Supplement: Supplementary file 1 — Additional file 1. Table S1. The list of diseases and genes in Neoseq project. Table S2. Results of Neoseq in the infants with IEM after TMS screening. [file 13023_2021_2116_MOESM1_ESM.docx]

**Supplementary table 1. The list of diseases and genes in Neoseq project**

| Disease | Gene | Inheritance | Presence of the gene in the traditional TMS-NGS screening. |
| --- | --- | --- | --- |
| Methylmalonic aciduria, MMA | *MMAA* | AR | Y |
|  | *MMAB* | AR | Y |
|  | *MMUT* | AR | Y |
|  | *MMADHC* | AR | Y |
|  | *MMACHC* | AR | Y |
|  | *LMBRD1* | AR | Y |
|  | *ABCD4* | AR | Y |
|  | *HCFC1* | XLR | Y |
| Propionic acidemia, PA | *PCCA* | AR | Y |
|  | *PCCB* | AR | Y |
| Glutaric acidemia I, GA1 | *GCDH* | AR | Y |
| Holocarboxylase synthetase deficiency | *HLCS* | AR | Y |
| Biotinidase deficiency | *BTD* | AR | Y |
| 3-Hydroxy-3-Methylglutaryl-Coenzyme A Lyase deficiency | *HMGCL* | AR | Y |
|  | *HMGCS2* | AR | Y |
| Isovaleric acidemia, IVA | *IVD* | AR | Y |
| 3-Methylcrotonyl-coenzyme A carboxylase deficiency, MCCD | *MCCC1* | AR | Y |
|  | *MCCC2* | AR | Y |
| Malonyl-CoA decarboxylase deficiency, MCD deficiency | *MLYCD* | AR | Y |
| 3-methylglutaconic aciduria type I | *AUH* | AR | Y |
| Ethylmalonic encephalopathy, EE | *ETHE1* | AR | Y |
| 2-methylbutyryl-CoA dehydrogenase deficiency | *ACADSB* | AR | Y |
| Isobutyryl-CoA dehydrogenase deficiency, IBDD | *ACAD8* | AR | Y |
| Maple syrup urine disease, MSUD | *BCKDHA* | AR | Y |
|  | *BCKDHB* | AR | Y |
|  | *DBT* | AR | Y |
|  | *DLD* | AR | Y |
|  | *FAH* | AR | Y |
| Tyrosinemia | *TAT* | AR | Y |
|  | *HPD* | AR | Y |
| Hyperphenylalaninemia，HPA | *PAH* | AR | Y |
|  | *PCBD1* | AR | Y |
|  | *PTS* | AR | Y |
|  | *QDPR* | AR | Y |
|  | *GCH1* | AR | Y |
|  | *SPR* | AR | Y |
| Hyperhomocysteinemia, HCY | *CBS* | AR | Y |
|  | *MTHFR* | AR | Y |
|  | *MTRR* | AR | Y |
|  | *MTR* | AR | Y |
| Non-ketotic hyperglycinemia, NKH | *GLDC* | AR | Y |
| Primary hypermethioninemia | *MAT1A* | AR/AD | Y |
| Argininemia | *ARG1* | AR | Y |
| Argininosuccinic aciduria | *ASL* | AR | Y |
| Carbamoyl phosphate syntetase I deficiency | *CPS1* | AR | Y |
| N-acetylglutamate synthase deficiency, NAGSD | *NAGS* | AR | Y |
| Ornithine transcarbamylase deficiency | *OTC* | XLR | Y |
| Citrullinemia type Ⅰ, CTLN1 | *ASS1* | AR | Y |
| Citrin deficiency | *SLC25A13* | AR | Y |
| Hyperornithinemia-hyperammonemia-homocitrullinuria syndrome, HHHS | *SLC25A15* | AR | Y |
| Ornithine transcarbamylase deficiency | *OAT* | AR | Y |
| Very long-chain acyl-CoA dehydrogenase deficiency, VLCAD | *ACADVL* | AR | Y |
| Long-chain 3-hydroxyl-CoA dehydrogenase deficiency, LCHAD | *HADHA* | AR | Y |
| Mitochondrial trifunctional protein deficiency, MTPD |  | AR | Y |
|  | *HADHB* | AR | Y |
| Short chain 3-hydroxyacyl-CoA dehydrogenase deficiency, SCHADD | *HADH* | AR | Y |
| Medium chain acyl-CoA dehydrogenase deficiency, MCADD | *ACADM* | AR | Y |
| Short-chain acyl-CoA dehydrogenase deficiency, SCADD | *ACADS* | AR | Y |
| Glutaric acidemia II, GA2 | *ETFA* | AR | Y |
|  | *ETFB* | AR | Y |
|  | *ETFDH* | AR | Y |
| Beta-ketothiolase deficiency, BKD | *ACAT1* | AR | Y |
| Carnitine palmitoyltransferase II deficiency, CPTII | *CPT2* | AR | Y |
| Primary carnitine deficiency, PCD | *SLC22A5* | AR | Y |
| Carnitine palmitoyltransferase I deficiency, CPT1 deficiency | *CPT1A* | AR | Y |
| Carnitine-acylcarnitine translocase deficiency, CACT deficiency | *SLC25A20* | AR | Y |
| Glycogen storage disease | *G6PC* | AR | N |
|  | *SLC37A4* | AR | N |
|  | *GAA* | AR | Y |
|  | *AGL* | AR | Y |
|  | *PYGL* | AR | N |
| Galactosemia | *GALT* | AR | Y |
|  | *GALK1* | AR | Y |
|  | *GALE* | AR | N |
| Hereditary fructose intolerance, HFI | *ALDOB* | AR | Y |
| X-linked adrenoleukodystrophy, X-ALD | *ABCD1* | XLR | Y |
| Mucopolysaccharidosis | *IDUA* | AR | Y |
|  | *IDS* | XLR | Y |
|  | *GALNS* | AR | Y |
|  | *NAGLU* | AR | Y |
| Globoid cell leukodystrophy, GLD | *GALC* | AR | Y |
| Gaucher disease, GD | *GBA* | AR | Y |
| Fabry disease | *GLA* | XLD | Y |
| Niemann-Pick disease | *NPC1* | AR | Y |
|  | *NPC2* | AR | Y |
|  | *SMPD1* | AR | Y |
| Beta-thalassemia | *HBB* | AR | N |
| Hemophilia B，HB | *F9* | XLR | N |
| Glucose-6-phosphate dehydrogenase | *G6PD* | XLD | Y |
| Growth hormone deficiency, GHD | *GH1* | AR | N |
|  |  | AR | N |
|  |  | AD | N |
|  |  | AR | N |
|  | *GHRHR* | AR | N |
| Vitamin D-dependent rickets | *CYP27B1* | AR | N |
|  | *VDR* | AR | N |
| X-linked dominant hypophosphatemia, XLH | *PHEX* | XLD | N |
| Cartilage-hair hypoplasia, CHH | *RMRP* | AR | N |
| Spinal muscular atrophy，SMA | *SMN1* | AR | N |
| Congenital Hypothyroidism, CH | *PAX8* | AD | Y |
|  | *THRA* | AD | N |
|  | *THRB* | AD | N |
|  | *TSHB* | AR | Y |
|  | *TSHR* | AR | Y |
|  | *TG* | AR | Y |
|  | *TPO* | AR | Y |
|  | *DUOXA2* | AR | N |
|  | *DUOX2* | AR | Y |
| Crigler-najjar syndrome，CNS | *UGT1A1* | AR | N |
| Progressive familial intrahepatic cholestasis, PFIC | *ATP8B1* | AR | Y |
|  | *ABCB11* | AR | Y |
| Nephroblastoma, NB | *WT1* | AD | N |
| Retinoblastoma, RB | *RB1* | AD | N |
| Non-syndromic hearing loss, NSHL | *GJB2* | AR | N |
|  | *GJB3* | AD | N |
|  | *SLC26A4* | AR | N |
|  | *MT-RNR1* |  | N |
|  | *MT-TH* |  | N |
| Mitochondrial encephalomyopathy, lactic acidosis and stroke-like episodes，MELAS | *MT-TL1* |  | N |
|  | *MT-ND5* |  | N |
| Severe combined immune deficiency, SCID | *ADA* | AR | N |
|  | *IL2RG* | XLR | N |
|  | *IL7R* | AR | N |
|  | *JAK3* | AR | N |
|  | *RAG1* | AR | N |
|  | *RAG2* | AR | N |
|  | *BCL11B* | AD | N |
| Autoimmune enteropathy, AIE | *FOXP3* | XLR | N |
| X-linked recessive chronic granulomatous disease, X-CGD | *CYBB* | XLR | N |
| X-linked agammaglobulinemia, XLA | *BTK* | XLR | N |
| Wiskott-Aldrich syndrome, WAS | *WAS* | XLR | N |
| Dihydropyrimidine dehydrogenase deficiency | *DPYD* | AR | N |
| Purine drug toxicity | *TPMT* | AR | N |
|  | *NUDT15* | AD | N |
| Inborn errors bileacid synthesis, IEBAS | *HSD3B7* | AR | N |
| Hepatolenticular degeneration, HLD | *ATP7B* | AR | Y |
| Congenial disordersod glycosylation, CGD | *PMM2* | AR | N |
| Sitosterolemia | *ABCG5* | AR | N |
|  | *ABCG8* | AR | N |

Y: Yes. N: No.

**Supplementary table 2. Results of Neoseq in the infants with IEM after TMS screening**

| **Case** | **TMS-NGS(panel)** | | | | | **Neoseq** | | | | **Consistency** |
| --- | --- | --- | --- | --- | --- | --- | --- | --- | --- | --- |
|  | **Disease** | **NBS result** | **Gene** | **Variant** | **Type** | **Disease** | **Gene** | **Variant** | **Type** |  |
| TP001 | MMA | C0=2.26  C3=7.73  C3/C2=1.39 | *MMUT* | c.323G>A  c.729-730insTT | P  P | MMA | *MMUT* | c.323G>A  c.729-730insTT | P  P | Y |
| TP002 | MMA | C3=9.32  C3/C2=0.72 | *MMACHC* | c.609G>A  c.658_660del | P  P | MMA | *MMACHC* | c.609G>A  c.658_660del | P  P | Y |
| TP003 | VLCADD | C12=0.91,C12:1=0.37,C14=3.32，C14:1=4.37，C14:2=0.61，C16:1=1.25 | *ACADVL* | c.1843C>T  c.1349G>A | LP  P | VLCADD | *ACADVL* | c.1843C>T  c.1349G>A | LP  P | Y |
| TP004 | 3MCCD | C4DC+C5OJ=8.64 | *MCCC1* | c.673C>T  c.161T>C | LP  US | undetected | *-* | - | - | N |
| TP005 | CTLN1 | CIT=86.04 | *ASS1*  *SLC22A5* | c.211-213del  c.694C>T  c.1400C>G | LP  LP  P | undetected | *SLC22A5* | c.1400C>G | P | N |
| TP006 | CTLN1 | CIT=299.58 | *ASS1* | c.1168G>A  c.1128_1134delinsG | P  LP | CTLN1 | *ASS1* | c.1168G>A | P | C |
| TP007 | CTLN1 | CIT=39.86 | *ASS1* | c.688+49T>C；  c.896C>A | US  US | undetected | - | - | - | N |
| TP008 | IHMET | MET=59.68 | *MATA1* | c.274T>C  c.268A>G | P  US | undetected | - | - | - | N |
| TP009 | IHMET | MET=101.86 | *MATA1* | c.242G>A  c.529C>T | US  LP | IHMET | *MATA1* | c.242G>A  c.529C>T | US  LP | Y |
| TP010 | IHMET | MET=265.15 | *MATA1* | c.91+1G>C  c.812A>G | P  LP | IHMET | *MATA1* | c.812A>G | LP | C |
| TP011 | IHMET | MET=101.1 | *MATA1* | c.791G>A | P | IHMET | *MATA1* | c.791G>A | P | Y |
| TP012 | PCD | C0=7.54 | *SLC22A5* | c.1400C>G* | P | PCD | *SLC22A5* | c.1400C>G* | P | Y |
| TP013 | PCD | C0=5.77 | *SLC22A5* | c.1400C>G  c.51C>G | P  P | PCD | *SLC22A5* | c.1400C>G  c.51C>G | P  P | Y |
| TP014 | PCD | C0=6.55 | *SLC22A5*  *ACADM* | SLC22A5: c.1400C>G  c.865C>T  ACADM: c.449_452del | P  P  P | PCD | *SLC22A5*  *ACADM* | SLC22A5: c.1400C>G  c.865C>T  ACADM: c.449_452del | P  P  P | Y |
| TP015 | PCD | C0=6.31 | *SLC22A5* | c.51C>G* | P | PCD | *SLC22A5* | c.51C>G* | P | Y |
| TP016 | PCD | C0=5.93 | *SLC22A5* | c.1400C>G  c.51C>G | P  P | PCD | *SLC22A5* | c.1400C>G  c.51C>G | P  P | Y |
| TP017 | BH4D | PHE=2431.38 | *PTS* | c.155A>G  c.259C>T | P  P | BH4D | *PTS* | c.155A>G  c.259C>T | P  P | Y |
| TP018 | CD | CIT=465.16，ARG=116.79 | *SLC25A13* | IVS16ins3Kb *  hom | P | undetected | *-* | - | - | N |
| TP019 | CD | CIT=86.27 | *SLC25A13* | c.852_855del  c.1638_1660dup | P  P | CD | *SLC25A13* | c.852_855del  c.1638_1660dup | P  P | Y |
| TP020 | CD | CIT=407.98，ARG=118.96 | *SLC25A13* | IVS16ins3Kb  c.1474C>T | P  US | undetected | *-* | - | - | N |
| TP021 | CD | CIT=53.2 | *SLC25A13* | c.852_855delTATG  IVS16ins3kb | P  P | CD | *SLC25A13*  *SLC26A4* | c.852_855delTATG  c.919-2A>G(INT) | P  P | C |
| TP022 | GA1 | C5DC+C6OH=3.26 | *GCDH* | c.109_110delCA  c.416C>G | P  P | GA1 | *GCDH* | c.416C>G | P | C |
| TP023 | GA1 | C5DC+C6OH=3.4 | *GCDH* | c.261_506-  433delinsATA  c.892G>A | LP  LP | GA1 | *GCDH* | c.892G>A | LP | C |
| TP024 | GA1 | C5DC+C6OH=3.9  C0=9.86 | *GCDH* | c.1244-2A>C  c.1235C>A | P  LP | GA1 | *GCDH* | c.1244-2A>C  c.1235C>A | P  LP | Y |
| TP025 | SCADD | C4=2.32 | *ACADS*  *ARG1* | ACADS:c.312G>C  c.322G>A  ARG1:c.922C>T | LP  P  US | SCADD | *ACADS* | c.312G>C  c.322G>A | LP  P | Y |
| TP026 | SCADD | C4=1.15 | *ACADS* | c.164C>T  c.1031A>G | P  P | SCADD | *ACADS* | c.164C>T  c.1031A>G | P  P | Y |
| TP027 | SCADD | C4=1.63 | *ACADS* | c.322G>A  c.1031A>G | P  P | SCADD | *ACADS* | c.322G>A  c.1031A>G | P  P | Y |
| TP028 | MCADD | C6=0.46  C8=1.09  C10=0.36  C10:1=0.25 | *ACADM* | c.469G>A  c.1136A>G | US  US | MCADD | *ACADM* | c.469G>A | US | C |
| TP029 | MCADD | C6=0.4  C8=0.88  C10=0.3  C10:1=0.21 | *ACADM*  *PTS* | ACADM:c.850-2A>G  c.1132G>A  PTS:c.84-291A>G | P  US  P | undetected | *PTS* | c.84-291A>G | P | N |
| TP030 | MCADD | C6=0.72  C6DC=0.31  C8=4.39  C10=0.34  C10:1=0.47 | *ACADM*  *ACADS* | ACADM:c.799G>A  c.1085G>A  ACADS: c.1054G>A | P  P  P | MCADD | *ACADM*  *ACADS* | ACADM:c.799G>A; c.1085G>A  ACADS: c.1054G>A | P  P  P | Y |
| TP031 | PKU | PHE=162.64  PHE/TYR=2.1 | *PAH* | c.728G>A  c.104T>C | P  LP | PKU | *PAH* | c.728G>A | P | C |
| TP032 | PKU | PHE=299.93  PHE/TYR=4.26 | *PAH* | c.1315+6T>A  c.1197A>T | P  P | PKU | *PAH* | c.1315+6T>A  c.1197A>T | P  P | Y |
| TP033 | PKU | PHE=188.89  PHE/TYR=1.76 | *PAH* | c.1139C>T  c.728G>A | P  P | PKU | *PAH* | c.1139C>T  c.728G>A | P  P | Y |
| TP034 | PKU | PHE=305.45  PHE/TYR=3.26 | *PAH* | c.940C>A  c.1076C>T | P  LP | PKU | *PAH* | c.940C>A  c.1076C>T | P  LP | Y |
| TP035 | ASA | CIT=155.16 | *ASL* | c.706C>T  c.-44+41_12+5del | P  P | undetected | - | - | - | N |
| TP036 | BKTD | C3DC+C4OH=3.58  C4DC+C5OH=1.31  C5:1=0.31 | *ACAT1*  *SLC25A20*  *HMGCL* | ACAT1: c.721dup  c.928G>C  SLC25A20: c.804del  HMGCL: c.799C>T | LP  LP  LP  US | BKTD | *ACAT1* | c.928G>C | LP | C |

TMS-NGS(panel): NGS followed Tandem Mass Spectrometry

P: Pathogenic. LP: Likely pathogenic. US:Uncertain significance.

Y: Yes. N: No. C:Carried.

PCD: Primary carnitine deficiency

PKU: Phenylketonuria

SCADD: Short-chain acyl-CoA dehydrogenase deficiency

MMA: Methylmalonic aciduria

IHMET: Isolated hypermethioninemia

VLCADD: Very long-chain acyl-CoA dehydrogenase

CD: Citrin Deficiency

CTLN1: Citrullinemia type I

BH4D: Tetrahydrobiopterin deficiency

MCADD: Medium-chain acyl-CoA dehydrogenase deficiency

GA1: Glutaric acidemia I

3MCCD: 3-methylcrotonyl-coenzyme A carboxylase deficiency

ASA: argininosuccinic aciduria

BKTD:β-ketothiolase deficiercy

CH: Congenital Hypothyroidism:

MELAS: Mitochondrial encephalomyopathy with lactic acidosis and stroke-like episodes

G6PD: Glucose-6-phosphate dehydrogenase
